# Polycystic Ovary Syndrome and NC-CAH: Distinct Characteristics and Common Findings. A Systematic Review

**DOI:** 10.3389/fendo.2019.00388

**Published:** 2019-06-19

**Authors:** Georgios Papadakis, Eleni A. Kandaraki, Ermioni Tseniklidi, Olga Papalou, Evanthia Diamanti-Kandarakis

**Affiliations:** ^1^STEPS Stoffwechselzentrum, Biel, Switzerland; ^2^Department of Endocrinology and Diabetes, HYGEIA Hospital, Athens, Greece

**Keywords:** non-classic adrenal hyperplasia, NC-CAH polycystic ovary syndrome, PCOS, 17-hydroxyprogesterone, 17-OHP, 21- hydroxylase deficiency, 21-OHD

## Abstract

**Background:** Twenty-one-hydroxylase–deficient non-classic adrenal hyperplasia (NC-CAH) is a very common autosomal recessive syndrome with prevalence between 1:1,000 and 1:2,000 individuals and the frequency varies according to ethnicity. On the other hand, polycystic ovary syndrome has a familial basis and it is inherited under a complex hereditary trait. This syndrome affects 6 to 10% of women in reproductive age and it is the most common endocrine disorder in young women. Our aim was to investigate, through a systematic review, the distinct characteristics and common findings of these syndromes.

**Methods:** The search period covered January 1970 to November 2018, using the scientific databases PubMed. Inclusion criteria were adult women patients with PCOS or NC-CAH. Search terms were “polycystic ovary syndrome,” “PCOS,” “non-classical adrenal hyperplasia,” “NC-CAH,” “21-hydroxylase deficiency.” From an initial 16,255 titles, the evaluations led to the final inclusion of 97 papers.

**Results:** The clinical features of NC-CAH are hirsutism and ovulatory and menstrual dysfunction therefore; differentiation between these two syndromes is difficult based on clinical grounds only. Additionally, NC-CAH and PCOS are both associated with obesity, insulin resistance, and dyslipidaemia. Reproductive abnormalities are also common between these hyperandrogenemic disorders since in patients with NC-CAH polycystic ovarian morphology and subfertility are present as they are in women with PCOS. The diagnosis of PCOS, is confirmed once other disorders that mimic PCOS have been excluded e.g., conditions that are related to oligoovulation or anovulation and/or hyperandrogenism, such as hyperprolactinaemia, thyroid disorders, non-classic congenital adrenal hyperplasia, and androgen-producing neoplasms.

**Conclusions:** The screening tool to distinguish non-classic adrenal hyperplasia from PCOS is the measurement of 17-hydroxyprogesterone levels. The basal levels of 17-hydroxyprogesterone may overlap, but ACTH stimulation testing can distinguish the two entities. In this review these two common endocrine disorders are discussed in an effort to unveil their commonalities and to illuminate their shadowed distinctive characteristics.

## Introduction

### Rationale

Twenty-one-hydroxylase–deficient non-classic adrenal hyperplasia (NC-CAH) is a relatively common autosomal recessive disorder with prevalence between 1: 1000 and 1: 2000 individuals and the frequency varies by ethnicity (1). On the other hand, polycystic ovary syndrome has a familial predisposition and is inherited under a complex genetic mechanism. Polycystic ovary syndrome affects 6 to 10% of reproductive-aged women and is one of the most common endocrine disorders (2). The clinical features of NC-CAH are hirsutism and ovulatory and menstrual dysfunction as well as insulin resistance and polycystic ovarian morphology (3).

### Objectives

Women with NC-CAH present with similar symptoms as with PCOS women and therefore, it is difficult to differentiate the two disorders based on clinical grounds solidly (4). This difficulty becomes apparent on several studies from different parts of the world, as in Turkey (5, 6), and in Greece (7), where women with NC-CAH were diagnosed as PCOS women at the beginning. About 1–4% of women in USA with clinical signs of androgen excess have NC-CAH (8–10). The main objective of this review is to find the common characteristics of the two syndromes.

### Research Question

The main search question of this review is to underline the main differences of the two syndromes and to discuss the methods to differentiate them.

## Methods

This review was performed following the preferred reporting items for systematic reviews and meta-analyses (PRISMA) guidelines (11).

### Criteria for Selection

Articles describing the main characteristics of PCOS and NC-CAH were considered, including case-control, cross-sectional, and cohort studies.

### Search Strategy

Studies in English that met the above criteria were collected by searching the Pubmed database: The MeSH terms (“polycystic ovary syndrome” OR “PCOS”) AND (“non-classical adrenal hyperplasia” OR “NC-CAH” OR “21- hydroxylase deficiency” OR “21-OHD”), complemented with manual searching, for publications listed up until November 2018. Two independent investigators conducted the searches. The list of recognized articles was scanned, and the reference lists of all related reviews and main articles were searched manually for more references. To decrease bias, two authors conducted the searches independently, and any disagreement between them was debated in a group discussion until a consensus was achieved.

### Study Selection

The authors independently assessed the titles and abstracts of all identified studies. Full reports were obtained for those studies that appeared to meet the inclusion criteria.

### Data Extraction and Management

The following data were recorded from each study: authors, country of origin, study type, the main outcome measures, and the outcomes.

### Inclusion and Exclusion Criteria

For further review, the authors screened abstracts and titles. Since every screened study was included in this systematic review, the researchers attempted to evaluate the relationship between PCOS and NC-CAH and underline their common findings and their differences. Studies on non-human creatures (i.e., animal studies), those published in languages other than English, those that were meta-analyses or systematic considerations, and those that presented insufficient data or were duplicate publications were also excluded. The research was conducted in conformity with the ethical standards of the field.

## Results

**Figure 1:**
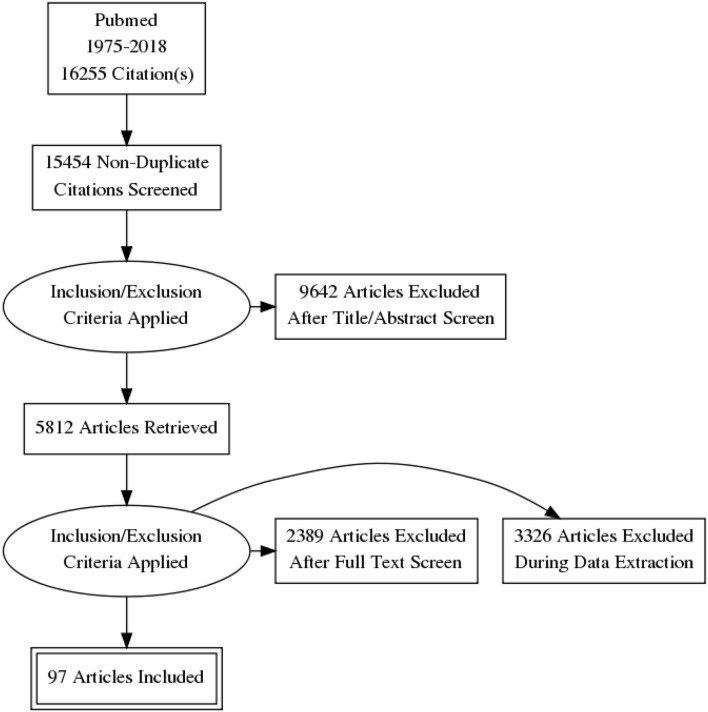
Flow diagram showing an overview of the articles selection process.

A total of 16,255 papers were initially identified. Of these, 801 were excluded because they were duplicates or had irrelevant contents. A total 9,642 more articles were excluded after titer and abstract screening and 5,812 articles were retrieved. A total of 5,715 articles were also excluded after full text screen and during data extraction. Finally, only 97 articles were included in this systematic review, as shown in Prisma Flow Diagram (Figure 1).

## Discussion

The main findings of our search were categorized according to epidemiology, genetics, pathophysiology, and clinical parameters. The main different characteristics and differentiated criteria were also described as well the different treatment options.

## Epidemiology of NC-CAH and PCOS

The NC-CAH is a common autosomal recessive disease, and the frequency changes in different ethnicities. The prevalence is as high as 1:1000 to 1:100 in white population (12–14), and even higher in women with Mediterranean, Hispanic or Eastern European Jew origin (8). Non-classical 21-hydroxylase deficiency (21-OHD) can be diagnosed by the elevation of 17-OHP that plot a nomogram between the range of unaffected persons and the levels of patients with the classical form of CAH. Similar to CAH, non-classical 21-OHD can cause premature growth of pubertal hair, acne, advanced bone age, accelerated linear growth velocity and reduced final height in both sexes (15). The classic form can be diagnosed in the neonatal screening test with a very high 17-hydroxyprogesterone (17-OHP) (16). It appears that the false negative rate is at least one-third in children with the moderate form of CAH. On the other hand, polycystic ovary syndrome (PCOS) appears in 6–10% of the women in reproductive years and it is the most common endocrine disorder in these women (2), but according to Rotterdam criteria, the prevalence can be as high as 10% (17).

## Distinct Characteristics

### Genetics

The etiology of PCOS remains equivocal; however, genetic, environmental and lifestyle factors interact each other and predispose to the syndrome. PCOS is considered to have a complex genetic background (2, 18, 19). The pathogenetic mechanisms of PCOS are connected to genetic and environmental parameters. An increased familial incidence implies that a complex genetic trait possible plays a role (20–23). Different genes are involved that are related to different hormonal and biochemical paths such as steroid and androgen synthesis, insulin production, folliculogenesis, gonadotropins, and weight control (24). Chromosomes such as chr 8 p 23.1, chr 11 p 14.1, and chr 9 q 22.32 have been associated with polycystic ovary syndrom, as well as chr 11p14.1 SNP, rs11031006 of the FSHB gene, have been directly associated with polycystic ovary syndrome and the synthesis of LH (19).

Nevertheless, NC-CAH is characterized by CYP21A2 deficiency, which is transmitted as an autosomal recessive disorder. The two CYP21A genes are both located in a 35-kilobase region of chromosome 6p21.3 within the major histocompatibility locus, and one gene is a non-functional pseudo-gene and the other an active gene. These two genes have a high degree of homology (up to 90%) and exchanges of DNA parts are often during meiosis. The exchange of small amounts of material can result in hybrid CYP21A1/CYP21A2 gene products. The enzyme activity is reduced at about 20–60%. A patient who is heterozygous for such mutations may have non-classic CYP21A2 deficiency.

Genetic testing is an alternative diagnostic tool for NC-CAH and it can be used when biochemical results are uncertain or when genetic counseling is necessary prior to conception. CYP21A2 genotyping should be performed to identify heterozygote carriers. On the other hand, there is no specific genetic test to determine the risk of developing PCOS in the female offspring or to diagnose the PCOS syndrome.

### Pathophysiology

In patients with CYP21A2 deficiency the cortisol synthesis is decreased because of the defective conversion of 17-OHP to 11-deoxycortisol. This biochemical path is determined by 21-hydroxylase and is defective because of mutation in the gene of CYP21A2. Only 20–60% of 21-hydroxylase enzyme activity is preserved. As a result, the corticotropin (ACTH) secretion is increased and that leads to increased adrenal stimulation and increased production of androgens. The enzyme activity in patients with non-classic form is reduced but remains sufficient to maintain the balance of glucocorticoid and mineralocorticoid production, at the expense of excessive androgen production.

On the other hand, PCOS has a puzzling pathophysiologic and biochemical basis (25), and genetic and environmental parameters interact with genetic factors generating aberrations related to metabolism and reproduction (26–28). The ovarian dysfunction is involved in the pathophysiology of polycystic ovary syndrome combined with hyperinsulinaemia and malfunction of the hypothalamic-pituitary-ovarian axis. The hypersecretion of luteinizing hormone (LH), which affects ovarian androgen production and oocyte development, as well as the insulin resistance at the peripheral tissues and the pancreatic β-cell dysfunction are also common features in PCOS. Moreover, the advanced glycation end- products (AGES) are involved in the pathogenic mechanisms of polycystic ovary syndrome. The chronic inflammation and the increased oxidative stress are related to AGES interaction and the reproductive and metabolic derangements of polycystic ovary syndrome (29, 30). Moreover, environmental toxins and endocrine disruptors, and in particular bispenol A, may predispose additional impacts on the syndrome as it has been shown, that they are related to metabolic and reproductive aberrations in PCOS (31, 32).

## Common Findings

### Hyperandrogenism

Hirsutism affects between 5 and 10% of women of reproductive age (33). Other common findings of hyperandrogonaemia are acne, alopecia, ovulatory dysfunction, and virilization and masculinization in most severe androgen excess. The virilization, the deepening of the voice and the clitoromegaly, are relatively rare findings and can be related to others sources of androgens hyperproduction, such as ovarian hyperthecosis or androgen-secreting neoplasms, although women with NC-CAH can more often present with minimal clitoromegaly (4).

PCOS is one of the most frequent causes of hyperandrogenism, with a prevalence of 50 to 80% of women with this sign (34, 35), whereas in NC-CAH it affects between 1 and 10% of these patients (34–37). Nevertheless, the frequency of PCOS is about 40–50 times higher than the frequency of NC-CAH in women in reproductive age or among women with hyperandrogonaemia (4).

Hirsutism constitutes the most common physical manifestation of hyperandrogenism (60–70%) in women with PCOS (38) and hyperandrogenaemia is the most characteristic hormonal alteration of PCOS. It has a multifactorial cause attributed mostly to the ovaries with a concomitant substantial contribution from the adrenals and a minor contribution from fatty tissue. Hyperandrogenism may be involved in deteriorating insulin resistance and in the concurrent obesity in women with polycystic ovary syndrome. In fact, androgen excess appears to participate as an additional factor, which deteriorates the cardio-metabolic profile of women with PCOS (39, 40).

Hirsutism can be milder in PCOS as they grow older, whereas, the prevalence of hirsutism increased in older women with NC-CAH and reaches about 90% in women over 40 years old. The degree of hirsutism does not differentiate between the two disorders neither in young nor in older ages.

### Polycystic Morphology

The PCOS morphology has been characterized by the presence of twelve or more follicles with a diameter of 2–9 mm, and/or an enlarged ovarian total volume of more than ten mL in one ovary, excluding a dominant follicle or a cyst (41). In PCOS polycystic ovaries on ultrasound are more common than in NC-CAH (70 vs. 40%) (3, 4). Polycystic ovaries are observed in about 75% in PCOS women (42). Similarly, 30–40% of patients with NC-CAH demonstrate polycystic ovarian morphology (PCO) (9, 43) with one other study suggesting higher percentage up to 82% (44).

Nevertheless, the ultrasound appearance of PCO is not a specific feature in PCOS syndrome. In the NIH 1990 diagnostic criteria, the PCOS was not considered a pathognomic criterion (45). Polycystic morphology was evident in 92% of female patients with hirsutism (46), in 87% of women presenting with symptoms of oligomenorrhea (46), in 26% of women presenting with symptoms of amenorrhea (46) and in 67% of women presenting with androgenic alopecia (47). Moreover, 82% of diabetic premenopausal female patients (48) and 40% of women with a history of gestational diabetes mellitus (49) have polycystic ovaries. Polycystic ovarian morphology up to 23% was also common in healthy women with regular menstrual cycles (50). Therefore, it is clear that PCO morphology by ultrasound is not a distinct characteristic between PCOS and NC-CAH its presence or absence does not serve any diagnostic outcome or further treatment choice.

### Ovulatory Disorders and Fertility

PCOS accounts for 70–90% of ovulatory disorders (51). Anovulation can present as frequent bleeding at intervals sooner than 21 days or non-frequent bleeding at intervals that arrive later than 35 days. Additionally, PCOS accounts for 70–90% of ovulatory disorders and consists the most frequent cause of ovulatory disorders (51). Moreover, about 50% of PCOS women present with primary infertility, and 25% with secondary infertility (52).

NC-CAH patients were less possible to present with anovulation, and more than 70% of women had a normal menstrual cycle and ovulation (24) and only 17% of NC-CAH women have menstrual irregularities (53). The subfertility in women with NC-CAH is milder and many women conceive spontaneously, the infertility may be up to 13%. Combined treatment for ovulation induction is also possible in these women. However, the different hormonal contribution of two conditions on ovulatory dysfunction may prove to be of major clinical significance on therapeutic decision, due to the specific role of glucocorticoids on NC-CAH (54).

### Pregnancy Complications

Women with polycystic ovary syndrome are at high risk of preterm delivery, pre-eclampsia and gestational diabetes during pregnancy (55). The spontaneous abortion rate in women with PCOS is 20–40% higher than healthy women (56). The connection between PCOS and gestational diabetes mellitus was initially supported by retrospective data (57). There is also a significant risk of spontaneous abortion in NC-CAH patients (25%) (58–60). Treatment with glucocorticoids seems to contribute to a decrease this risk (60). Most women with NC-CAH conceive with ovulation induction, with the treatment of glucocorticoids alone or combined with clomiphene citrate, and the rate of early pregnancy loss is equal to healthy women.

Genetic counseling in women with NC-CAH is also useful. Genotyping should be suggested in patients who seek fertility because approximately two-thirds of patients with non-classic 21-OHD carry a severe mutation. The total risk of severe (classic) 21-OHD in an offspring of a patient with the non-classic type is about 2.5%, whereas the risk of non-classic deficiency is ~15%. Knowledge of the father's genotype can help assess these risks more precisely (61, 62). Therefore, the distinction between PCOS and NC-CAH is mandatory for genetic counseling on several aspects including future generations.

### Metabolic Parameters

Metabolic syndrome encompasses a mixture of: increased insulin resistance, high lipids level, increased cardiovascular risk, and increased central obesity. Women with polycystic ovary syndrome are also at high risk of having metabolic syndrome (63).

Obesity is more common in women with polycystic ovary disease than other hyperandrogenic syndromes (3, 10) and the percentage of central obesity among women with PCOS ranges between 20 and 85.5% (64). Nevertheless, NC-CAH is also related to increased obesity, up to 41% (65).

Patients with polycystic ovary syndrome have also a higher risk for diabetes mellitus type 2. The prevalence of insulin resistance has been noted in 60–80% of obese women with PCOS (66, 67). Earlier studies reported a prevalence of impaired glucose tolerance of 35% and diabetes mellitus type 2 of 10% in female patients presenting with PCOS (68, 69). Moreover, affected women have noticeable insulin resistance, which is irrelevant of obesity (70).

Insulin resistance is very common characteristic in PCOS women, found up 60–80% in lean and 95% in obese women with the syndrome (66). Insulin resistance is described as the condition in which a cell, tissue or organism needs above-normal amounts of insulin to respond normally to a certain glucose load. It is related with increased insulin secretion by pancreatic β-cells and compensatory hyperinsulinemia, whereas the blood glucose remains normal. Insulin resistance can be calculated by HOMA (Homeostasis Model Assessment) INDEX (product of fasting plasma insulin [mU/L] and glucose [mmol/L] concentrations divided by 22.5). Insulin resistance itself is connected to altered large artery compliance and endothelial function. Insulin resistance may be more severe, but probably not more common in polycystic ovarian syndrome than in NC-CAH (3, 4, 65, 71). The degree of hyperinsulinemia was higher in female patients with PCOS and central obesity, whereas in lean women with PCOS the metabolic abnormalities equal often and severe as in female patients with non-classic adrenal hyperplasia (53).

The hyperinsulinemia contributes to the augmented production of androgens by the adrenal glands (72) and the ovaries. This is achieved with the activation of P450c17a (CYP17 mRNA and protein expression) which increases the effect of the CYP21A deficiency and so he steroidogenic precursors are diverted to the path of androgen production (73). Moreover, hyperinsulinaemia obstructs liver production of SHGB, and so the testosterone availability increases.

Women with PCOS are at higher risk for impaired glucose tolerance and diabetes mellitus type 2 compared to healthy women (69, 74, 75). A diagnosis of PCOS increases the risk of developing type 2 diabetes mellitus up to 5- to 10-fold (69, 74, 75). The overall prevalence of glucose intolerance in PCOS was 30–35, and 3–10% for type two diabetes mellitus, depending on the degree of obesity as well as ethnicity. Non-obese female patients with PCOS had a 10–15% prevalence of impaired glucose tolerance and a 1–2% prevalence of type two diabetes mellitus (69, 74, 75).

About 70% of PCOS patients present abnormal serum lipid levels such high low-density lipoprotein (LDL) and triglyceride levels and low high-density lipoprotein (HDL) levels (28). The lipids elevation is regardless of body mass index (BMI) (18, 76). Moreover, hypercholesterolemia is also common in women with NC-CAH (up to 46%) (65).

### Cardiovascular Risk

PCOS incorporates many metabolic abnormalities that result in high risk for cardiovascular diseases. The metabolic aberrations include the impaired glucose tolerance, the dyslipidaemia, the low-grade inflammation and the increased coagulation factors (10, 63). Moreover, the active AGE-RAGE axis contributes to the atherosclerosis as well as the endothelial dysfunction (77). Although all these predisposing factor to cardiovascular disease it remains uncertain whether they result in a higher mortality rate (78, 79).

On the other hand, the same cardiovascular factors are met also in NC-CAH women. Patients with NC-CAH present also very often with obesity, insulin resistance and higher lipids level. However, it remains uncertain whether women with NC-CAH have actually a higher cardiovascular risk and higher mortality when compared with female patients without hyperandrogonaemia (24).

### Mood Disturbances

Women with PCOS present increased risk for depression and this is independent of androgen levels, hirsutism, acne, obesity, and infertility (80). The prevalence of depression in PCOS women in different studies is high reaching up to 64% (81–83). Likewise, depression and anxiety symptoms are frequent in female patients with NC-CAH, up to 50% (84). Therefore, it is important to monitor the patient's mood symptoms during treatment. Some studies suggest that treatment of the hirsutism can improve quality of life and reduce depression and anxiety symptoms (85, 86).

### Hormonal and Biochemical Parameters

A high ratio of luteinizing hormone (LH) to follicle-stimulating hormone (FSH) can be found in PCOS women, as well in women with NC-CAH but to a lesser extend (4, 43).

Patients with PCOS and NC-CAH do not differ in their hormonal parameters. The testosterone levels are elevated in both syndromes often to a similar degree (4, 43) or can be higher in NC-CAH women (3, 10). The DHEAS levels can be equally elevated in both syndromes (43, 87), or can be higher in NC-CAH (3, 4, 10).

The Table 1 summarizes the main differences and common characteristics of the two syndromes.

**Table 1:** Common and different characteristics of the two syndromes.

	**NC-CAH**	**PCOS**
**Prevalence**	**Rare syndrome**	**Common syndrome**
Prevalence in reproductive age women (4)	0.1–0.05%	4–6%
Prevalence in hyperandrogenic patients (4)	1–10%	50–80%
Difference in prevalence according to ethnicity	Major differences High-risk group: women with Ashkenazi Jewish, Hispanic, and Mediterranean origin	Only minor differences
**Pathophysiology**	**Defective enzymatic activity**	**Genetic and environmental factors**
**Hyperandrogenaemia manifestations**	**Common**	**Common**
Hirsutism	Common (59%)	Common (60–70%)
Acne (71, 88, 89)	Common (33%)	Common (14–25%)
Clinical presentation of hirsutism as woman gets older	Similar or increase	Milder
**Gynecological problems**	**Common**	**More common**
Menstrual irregularities (53)	Common (17%)	Very common (90%)
Polycystic ovaries (3, 71)	Common (40%)	Very common (70%)
Infertility (52)	Yes, milder (13%)	Yes (25–50%)
Pregnancy complications (56, 58–60)	Yes, spontaneous abortions: common (25%)	Yes, spontaneous abortions: common (20–40%)
**Metabolic aberrations**	**Common**	**More common, more severe**
Type 2 diabetes mellitus (35, 69, 74, 75)	<4%	3–10%
Obesity (10, 65, 67)	Common (12.2–41%)	Very common (28.4–85%)
Insulin resistance (65, 66)	Common (29%)	Very common, more severe (60–80%)
Dyslipidaemia (28, 65)	Common (46%)	Very common (70%)
**Mood disorders/depression** **(****81****–****84****)**	**Common (50%)**	**Common (21–64%)**
**Inheritance mechanism** **(****4****)**	**Autosomal recessive**	**Unclear**
**Special test for differential diagnosis**	**Yes**	**Exclusion of other conditions**
Basal 17-OHP >2 ng/mL (53)	87%	25%
Specific Hormonal diagnosis (4)	ACTH-stimulated 17-OHP	None
**Other test**		
LH/FSH >2 (53)	Not very common (9%)	Common (22–29%)
DHEAS (53)	Elevated or very elevated	Elevated
Testosterone (53)	Elevated	Equally elevated
**Therapy options**	**OCS, glucocorticoids, antiandrogens, clomiphene citrate**	**OCS, weight loss, antiandrogens, metformin, clomiphene citrate**

In conclusion and clearly PCOS is a more common syndrome. The pathophysiology of PCOS is multifactorial, whereas in NC-CAH there is a clear mechanism of a defective enzymatic activity duo to specific genes. Moreover, PCOS present more often with the metabolic and gynecological aberrations, although these are also present with different degree of frequency in NC-CAH women.

## Differential Diagnosis

According to the 1990 National Institute of Health (NIH) criteria, the existence of oligoovulation and/or anovulation and clinical and/or biochemical indication of hyperandrogenaemia are necessary, irrespective of the existence of polycystic ovaries on U/S examination (43). The 2004 Rotterdam criteria proposed that PCOS should be defined when at least two of the three aforementioned criteria exist, and other diseases or disorders that can resemble the polycystic ovary syndrome can be excluded (90). Among these are the thyroid disease, hyperprolactinemia, and non-classic congenital adrenal hyperplasia. In women with more severe phenotypes, a further evaluation is necessary in order to exclude other causes, such as Cushing's syndrome and acromegaly. Based on the Androgen Excess PCOS Society Criteria (AE-PCOS Society Task Force) PCOS should be defined when the following are present: hyperandrogenaemia (clinical and/or biochemical), ovarian dysfunction (oligo-anovulation and/or polycystic ovarian morphology), while related disorders are excluded (91). Table 2 summarizes the different diagnostic criteria for PCOS.

**Table 2:** Summarizes the different diagnostic criteria for PCOS.

**A comparison of diagnostic criteria for polycystic ovary syndrome**
1990 National Institute of Child Health and Human Development (NICHD) diagnostic criteria: 1. Clinical and/or biochemical signs of hyperandrogenism 2. Oligo- or chronic anovulation Other reasons for androgen excess and annovulatory infertility are excluded
2003 European Society for Human Reproduction and Embryology and American Society for Reproductive Medicine (ESHRE/ASRM or Rotterdam) Criteria: 1. Oligo- or chronic anovulation 2. Clinical and/or biochemical signs of hyperandrogenism 3. Polycystic ovaries Other reasons for androgen excess and annovulatory infertility are excluded
2006 Androgen Excess Society (AES) crieteria: 1. Hirsutism and/or hyperandrogenemia 2. Oligo-anovulation and/or polycystic ovaries Other reasons for androgen excess and annovulatory infertility are excluded

PCOS is 40 to 50 times more frequent than that of NC-CAH in reproductive aged women or among hyperandrogenic women (4). NC-CAH is uncommon in women of African-American and Hispanic-Puerto Rican origin (1, 71). Nevertheless, distinguishing NC-CAH from PCOS is recommended in all female patients of Eastern European Jewish origin (prevalence 1:27), and women of Hispanic (prevalence 1:40), Slavic (prevalence 1:50) or Italian origin (prevalence 1:300) (92). Nevertheless, it is suggested to distinguish NC-CAH from PCOS in all female patients with apparent PCOS. Instead the prevalence of PCOS according to ethnicity has a minor variation (93).

In women with hyperandrogonenism (hirsutism and/or acne) and oligomenorrhea the non-classic type of NC-CAH should be distinguished from polycystic ovary syndrome. PCOS is much more common than NC-CAH. The basal levels of 17-OHP may overlap, but ACTH stimulation testing can distinguish the two entities (53).

When the hormonal and biochemical results are borderline a genetic test for NC-CAH can be done. Genetic counseling is also needed prior to conception. When a parent has NC-CAH the risk for the child to develop (classic) 21-OHD is ~2.5%, while the risk of non-classic deficiency is ~15% (59). The risk can be more precisely assessed when testing the partner's genotype.

The prevalence of NC-CAH in women who present with PCOS-type picture depends on the population. High-risk groups include women with Mediterranean, Hispanic, and Ashkenazi Jewish origin. Testing for NC-CAH with measurement of a basal 17-OHP is recommended in populations of high risk, as well in all women who present with clinical picture compatible with PCOS (94).

A basal 17-OHP should be measured at around 8 am during the follicular phase of the cycle. For a woman with irregular or no menses a random blood sample can be drawn. A basal 17-OHP more than 200 ng/dL (6 nmol/L) is diagnostic for NC-CAH is strongly suggested and further evaluation is required, whereas a value <200 ng/dL (6 nmol/L) suggest that the diagnosis is unlikely. The ACTH stimulation test confirms the diagnosis.

When the basal 17-OHP is more than 6 nmol/L, a Synachten test should follow. A high dose of ACTH is used (250 mcg). The diagnosis of NC-CAH is confirmed when the 17-OHP reached or exceeds 1,500 ng/dL (43 nmol/L) after Synachten test (8, 9). When the stimulated values in 1 h are between 1,000 and 1,500 ng/dL (30 to 43 nmol/L) a genotyping is recommended to confirm the diagnosis.

The basal 17-OHP increases during the preovulatory or luteal phase of menstrual cycle. Therefore, the sample should be obtain within the 10 first days after the beginning of the menstruation or any time when the patient in amenorrheic. A serum progesterone can be also measured to exclude that the blood was not drawn during the luteal phase of the menstrual cycle. A serum progesteron >400 ng/dL (12.7 nmol/L) indicates a luteal phase.

About 20% of patients with PCOS have also elevated 17-OHP values. A value of 200 ng/ml might be suggestive of NC-CAH but in order to distinguish the two syndromes, it is suggested to perform an ACTH stimulation test for values <1,000 ng/ml in order to confirm the diagnosis of NC-CAH.

Almost two-thirds of patients with non-classic 21-OHD are compound heterozygotes, characterized by a severe or mild mutation on their alleles. Genotyping is therefore useful for patients who seek fertility (61, 62). Men's genotyping helps to assess the risk. Men with NC-CAH are asymptomatic. The criteria for diagnosis are the same as in women and the diagnosis is usually made for a family evaluation (95). When one patient has the non-classical form the risk for the child for classic 21-hydroxylase form is 2.5% and for the non-classic 15% (59).

## Approach to Treatment

The oral contraceptives and the anti-androgen therapy is the primary therapy treatment for the symptoms of hyperandrogonaemia in adult women with either PCOS or non-classic 21-OHD who are not pursuing fertility. The role of glucocorticoid therapy is more documented for NC-CAH but it may be used for hyperandrogenic symptoms and menstrual cycle management in women who do not take or tolerate oral contraceptives or antiandrogens, such as spironolactone, therapy.

The glucocorticoids reduce the androgen production from the adrenal glands by suppressing the corticotropin-releasing hormone (CRH) and the corticotropin (ACTH). Nevertheless, hydrocortisone and dexamethasone seem to be more effective than oral contraceptives for suppressing serum adrenal androgen concentrations but less effective for decreasing clinical apparent hirsutism. Moreover, the glucocorticoids even in mild excess have many potential risks and side effects (96).

Considering that oral contraceptives suppress ovarian and adrenal androgens and ACTH. They have been accepted as the first-line therapy in adults because they seem to be more effective for hirsutism than glucocorticoids. Glucocorticoids can be prescribed to women with NC-CAH who cannot tolerate or don't respond to or oral contraceptives and antiandrogen therapies.

Antiandrogens, such as spironolactone, are also effective, though antiandrogen monotherapy is not recommended because of possible teratogenicity. Oral contraceptives can be started and spironolactone can be added after 6 months if the cosmetic response with an oral contraceptive alone has not been adequate. Dexamethasone crosses the placenta and therefore is not suggested in sexually active women, and instead, hydrocortisone, prednisone, or prednisolone are preferred. Oral contraceptives are suggested instead of glucocorticoids for menstrual cycle management. Glucocorticoids can be used for ovulation induction in anovulary women who seek fertility, and clomiphene citrate can be also added.

The treatment of PCOS is based also in oral contraceptives as basic treatment for the disorders of menstruation and the clinical hirsutism and acne. The contraindications of oral contraceptives should be also taken into account. Oral contraceptives protect also the endometrium and offer contraception. When oral contraceptives are contraindicated a progestin pills or cyclical progestins can be provided. Exercise and diet can improve the metabolic parameters as well the reproductive dysfunction. Weight reduction is probable useful for the reproductive and metabolic disorders of women with PCOS, whereas weight reduction is probable unsatisfactory as a treatment for patients with normal body weight. Metformin is a second-line treatment for the regulation of metabolic parameters and menstrual irregularity. Metformin is recommended for women with PCOS who have type 2 diabetes mellitus or impaired glucose tolerance (IGT) who fail lifestyle modification. Pioglitazone has also been used in women with PCOS, providing more metabolic and reproductive benefits and possibly protection from developing diabetes and cardiovascular problem. Inositols are second messengers for insulin, and their deficiency contributes to the various features of PCOS and when given to PCOS women they can alleviate the metabolic, menstrual/ovulatory, and cutaneous hyperandrogenic features of the syndrome. Clomiphene citrate (or the estrogen modulator letrozole) can be used as the primary therapy for the infertility (94). Alternative options for infertility treatment in anovulatory women are the gonadotropins and the *in vitro* fertilization. Metformin is not suggested for ovulation induction, whereas the laparoscopic diathermy of the ovaries may be used under specific circumstances. The management of PCOS encompass a tailored approach to individual needs of each patient (97).

In both syndromes, the cardiometabolic alterations require regular screening and therefore statins and anti-obesity drugs can be helpful for the metabolic parameters. Bariatric surgery is recommended only in severe obese female patients with PCOS or NC-CAH. Hirsutism can be approached with cosmetic procedures in both PCOS and NC-CAH patients. For women with patient-important hirsutism possible solutions are direct hair removal and chemical depilatory agents that dissolve the hair. The plucking and waxing, are quite cheap and safe methods, although they can be unpleasant.

### Limitations

The strength of this systematic review is that we compared two syndromes an effort was made to illuminate their hidden characteristics. Clinicians should recognize their main differences and proceed to the necessary tests in order to differentiate them. This knowledge can be implemented in the clinical practice. Limitation of this study is that we used only one database, Pubmed, and only the English written articles.

## Conclusions

Women with NC-CAH due to 21-OHD and women with PCOS have similar clinical presentation, with hyperandrogenism, oligomenorrhea, and polycystic ovaries. Insulin resistance, hyperinsulinism, and polycystic ovarian morphology were all detected in a great number of NC-CAH women. PCOS is more common, but NC-CAH should be also excluded by measuring the serum 17-OHP during the first days of follicular phase (94).

NC-CAH and PCOS present with analogous clinical characteristics and augmented androgen levels. In NC-CAH the androgens are as high as in obese PCOS women, but the metabolic profile is similar to lean PCOS women. Women with PCOS present more often with oligomenorrhea or amenorrhea and polycystic ovarian morphology. Moreover, they present with a LH/FSH ratio more than 2:1 (53). The screening tool to distinguish non-classic adrenal hyperplasia from PCOS is the basal 17-OHP levels and the acute ACTH stimulation test. Genetic screening may also be necessary in difficult cases of PCOS and NC-CAH, when their commonalities on clinical and hormonal grounds, even unveiled cannot illuminate their shadowed distinctive characteristics.

## Author Contributions

All authors listed have made a substantial, direct and intellectual contribution to the work, and approved it for publication.

### Conflict of Interest Statement

The authors declare that the research was conducted in the absence of any commercial or financial relationships that could be construed as a potential conflict of interest.
